# The dual role of autophagy in HPV-positive head and neck squamous cell carcinoma: a systematic review

**DOI:** 10.1007/s00432-023-05514-3

**Published:** 2024-01-31

**Authors:** Sam Augustine Kandathil, Arian Akhondi, Lorenz Kadletz-Wanke, Gregor Heiduschka, Nikolai Engedal, Faris F. Brkic

**Affiliations:** 1 Department of Otorhinolaryngology, Head and Neck Surgery, Medical University of Vienna, Währinger Gürtel 18-20, 1090 Vienna, Austria; 2 Division of Anatomy, Center for Anatomy and Cell Biology, Medical University of Vienna, Vienna, Austria; 3 Department of Tumor Biology, Institute for Cancer Research, Oslo University Hospital, Oslo, Norway

**Keywords:** ATG, Autophagy, HNSCC, HPV, Radiosensitivity, Survival

## Abstract

**Purpose:**

Human papilloma virus (HPV)-positive head and neck squamous cell carcinoma (HNSCC) displays distinct epidemiological, clinical, and molecular characteristics compared to the negative counterpart. Alterations in autophagy play an important role in cancer, and emerging evidence indicates an interplay of autophagy in HNSCC carcinogenesis and tumor promotion. However, the influence of HPV infection on autophagy in HNSCC has received less attention and has not been previously reviewed. Therefore, we here aimed to systematically review the role of autophagy explicitly in HPV^+^ HNSCC.

**Methods:**

Studies accessible in PubMed, Embase, Scopus, and Web of Science investigating HNSCC, highlighting the molecular biological differences between HPV^−^ and HPV^+^ HNSCC and its influences on autophagy in HNSCC were analyzed according to the PRISMA statement. A total of 10 articles were identified, included, and summarized.

**Results:**

The HPV16 E7 oncoprotein was reported to be involved in the degradation of AMBRA1 and STING, and to enhance chemotherapy-induced cell death via lethal mitophagy in HNSCC cells. Autophagy-associated gene signatures correlated with HPV-subtype and overall survival. Additionally, immunohistochemical (IHC) analyses indicate that high LC3B expression correlates with poor overall survival in oropharyngeal HNSCC patients.

**Conclusion:**

HPV may dampen general bulk autophagic flux via degradation of AMBRA1 but may promote selective autophagic degradation of STING and mitochondria. Interpretations of correlations between autophagy-associated gene expressions or IHC analyses of autophagy-related (ATG) proteins in paraffin embedded tissue with clinicopathological features without biological validation need to be taken with caution.

**Supplementary Information:**

The online version contains supplementary material available at 10.1007/s00432-023-05514-3.

## Introduction

Head and neck squamous cell carcinoma (HNSCC) mainly arises from the mucosa of the oral cavity, pharynx, larynx, and nasal and paranasal sinuses. With a global incidence of 878 348 new registered cases, it was the seventh most common malignancy worldwide in 2020 (Sung et al. 2021). The region of tumor appearance is easily susceptible to exposition of different carcinogens, mostly nicotine and alcohol. Due to reduced exposure to noted risk factors in recent decades, the global incidence of HNSCC is steadily declining (Vigneswaran and Williams 2014; Ellington et al. 2020). Meanwhile, the incidence of human papillomavirus—positive (HPV^+^) HNSCC, particularly oropharyngeal squamous cell carcinoma (OPSCC), is slowly rising in recent decades (Johnson et al. 2020; Lechner et al. 2022). A recent meta-analysis reported the global percentage of OPSCC that are HPV^+^ to be 33% in 2021. However, high variation in the reported prevalence depending on the geographical region need to be considered, ranging from 0% in South India to 85% in Lebanon (Carlander et al. 2021). Widely used clinical risk factors in relation to HNSCC are the TNM classification system and HPV/p16 status. HPV types have been categorized regarding their potential to induce malignant transformation of epithelial cells of the cervix or benign epithelial lesions into ‘high-risk’ (HR) and ‘low-risk’ (LR) (Cohen et al. 2019). Among the HR-HPV types involved in head and neck carcinogenesis, HPV16 was reported to be the most common HPV type in OPSCC, with a prevalence over 80%, followed by HPV33 and HPV18 (0.7 and 0.3%) in a recent meta-analysis including studies from 44 countries (Ndiaye et al. 2014). Pathological workup can further yield insights into proliferation, vascularization, and perineural invasion. This data help clinicians define an appropriate therapy regimen within an interdisciplinary team (Huang and O’Sullivan 2017; Zanoni et al. 2019; Saidak et al. 2020).

Constant improvement with new guidelines and adjustments based on clinical studies, such as checkpoint inhibitors and robotic surgery are made to decrease mortality rates and to improve quality of life for HNSCC patients (Masarwy et al. 2021; Virgilio et al. 2021). Recently, modifications within the 8th edition of the American Joint Committee of Cancer (AJCC) staging system have acknowledged major differences between HPV^+^ and HPV^−^ HNSCC, and these are considered as two distinct entities with different tumor biology. This differentiation is based on different risk-, molecular-, and outcome-profiles (Lechner et al. 2022; Gillison et al. 2008). Although mostly linked to higher radiosensitivity and better clinical outcomes, a study from our center reported 15 (7.1%) cases with distant metastasis of a total 211 HPV^+^ OPSCC patients (Brkic et al. 2021). Importantly, clinical risk factors, typical for HPV^−^ HNSCC, are shown to have a low prognostic value for HPV^+^ patients (Brkic et al. 2021; Mendenhall et al. 2019).

**Fig. 1:**
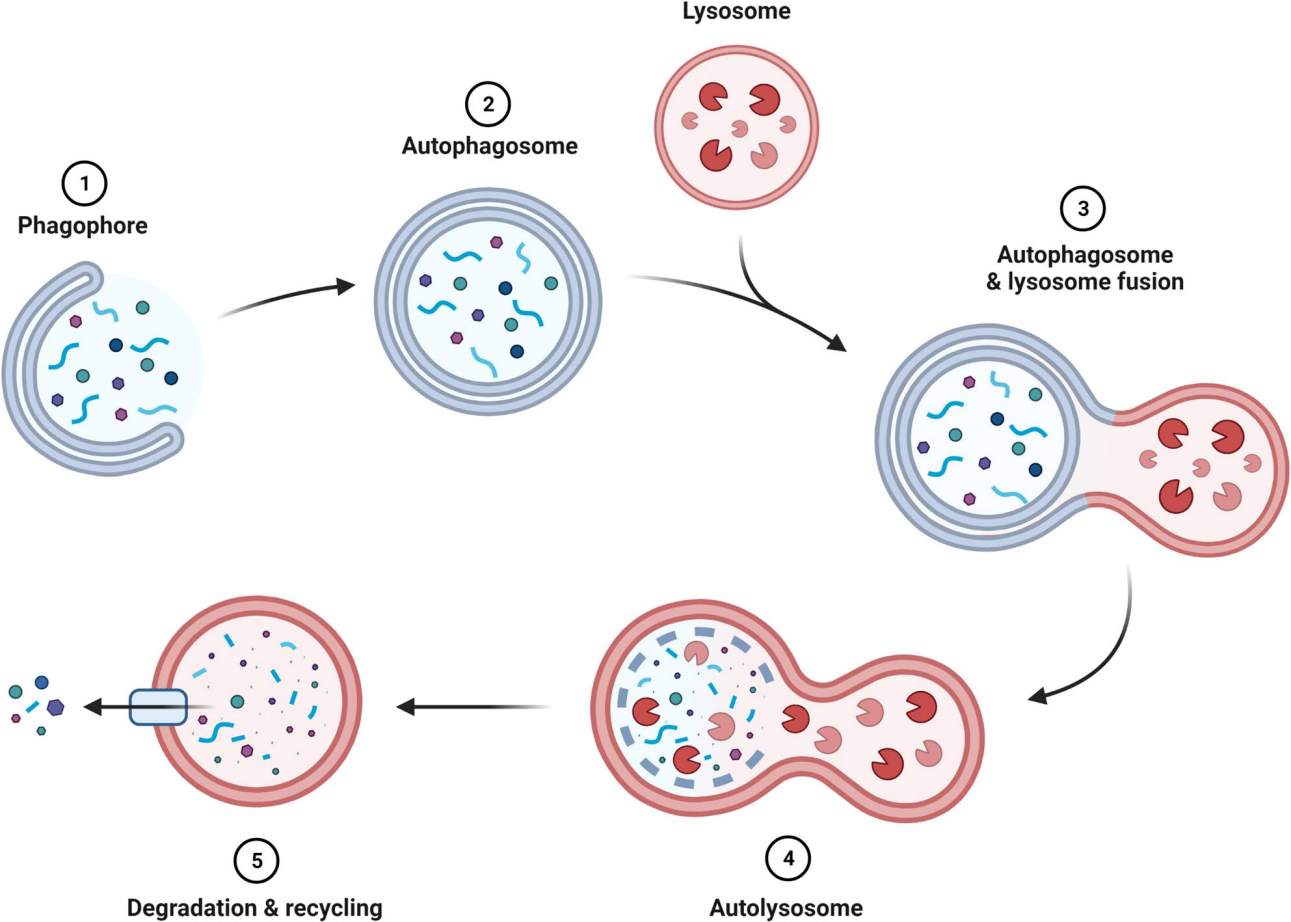
Schematic diagram of the autophagy machinery in mammalian cells

Macroautophagy, which will be referred to as “autophagy”, is a dynamic cellular recycling process through which cells can digest their own cellular contents by lysosomal degradation (Fig. 1). Autophagy substrates include single proteins to whole organelles, which are degraded to generate energy and new building blocks to facilitate cell survival and cellular renewal (Mizushima and Komatsu 2011). The substrates are targeted for degradation upon being surrounded and sequestered by an expanding membrane cisterna termed the “phagophore” (Seglen et al. 1987), which closes in on itself to form a sequestering double/multi-membrane vacuole termed the “autophagosome”. The cytoplasmic contents inside the autophagosomes are degraded upon fusion of the outer autophagosome membrane with a lysosome. Autophagosomes were initially found by electron microscopy studies of mouse and rat cells (Clark 1957; Novikoff 1959; Ashford and Porter 1962; Novikoff and Essner 1962; Duve and Wattiaux 1966; Yang and Klionsky 2010). Genetic studies in yeast, identified a set of autophagy-related (ATG) genes, which are evolutionary conserved from plants to humans, and which make up the core machinery of the autophagic pathway. Many of the ATGs, including ULK1 (homologue of yeast ATG1), ATG5, Beclin-1 (homologue of yeast ATG6) and ATG12, play important roles in autophagosome formation.

Autophagy may act preventive in the early stages of carcinogenesis (Galluzzi et al. 2015), whilst it can act to promote or limit the progression of established cancers in a manner that may be tumor- and context dependent (Gewirtz 2014; Zhong et al. 2016; Marsh et al. 2021; Galluzzi et al. 2017; Linder and Kögel 2019; Rojas-Sanchez et al. 2019; Towers et al. 2020). Data mining of the HNSCC cohort of the cancer genome atlas (TCGA) revealed that high mRNA expression of ATG5 or Beclin-1 (BECN1) is associated with decreased overall survival (New et al. 2017; Digomann et al. 2019), indicating a putative association between increased activity of autophagy and decreased overall survival. On the other hand, low protein expression of Beclin-1 has been linked to poor prognosis in hypopharyngeal (Wang et al. xxxx), laryngeal (Huang et al. 2013), and oral tongue HNSCC (Hu et al. 2016). Experimental studies of HNSCC cells have suggested that autophagy may promote treatment resistance through cell protective mechanisms in response to several types of chemotherapeutics or radiation (Sannigrahi et al. 2015). However, in some cases, autophagy can instead contribute to therapy-induced cell death (Sannigrahi et al. 2015).

Only few studies assessing the autophagic activity in HPV^+^ HNSCC could be identified. Research in this field might lead to better risk-stratification and new therapy options, which are of utmost interest for the subgroup of HPV^+^ HNSCC patients with dire prognosis. Although several reviews summarize recent findings on the roles of autophagy in HNSCC (Sannigrahi et al. 2015; Rikiishi 2012; Wu et al. 2015; Cosway and Lovat 2016; Adhauliya et al. 2016; Cruz-Gregorio et al. 2019; Harsha et al. xxxx; Liao et al. 2021; Raudenská et al. 2021; Bos et al. 2021; Anderson and O’Sullivan 2022), similar literature reviews giving an overview of previous studies looking specifically into autophagy in HPV^+^ HNSCC are still missing. Therefore, we here aimed to systematically review the role of autophagy explicitly in HPV^+^ HNSCC. This systematic review offers a new perspective on available evidence regarding the influence of HPV on autophagy activity and autophagy-associated genes in HPV^+^ HNSCC.

## Methods

### Search strategy, eligibility criteria, and data extraction

This systematic review was conducted in compliance with the Preferred Reporting Items for Systematic Reviews and Meta-Analyses (PRISMA) guidelines and recommendations (Supplementary Table I) (Page et al. 2021). All studies investigating HNSCC, highlighting the molecular biological differences between HPV^−^ and HPV^+^ HNSCC and its influences on autophagy in HNSCC regardless of study design, virus serotype, or animal model were included in this review. To ensure a sufficient and broad coverage of primary literature, suitable studies accessible in PubMed, Embase, Scopus, and Web of Science published until November 30, 2022, were included in the primary screening. We performed the literature search using a specific Boolean search combination, e.g., for PubMed research: *Autophagy AND HPV AND HNSCC.* This search paradigm was adapted accordingly, if necessary, to be suitable for the extraction of information using different databases.

Existing meta-analyses, reviews, and poster presentations were not considered for this review. Moreover, only original articles written in English were included in this review.

Primary screening of titles and abstracts of suitable studies found with the search paradigm across the four databases was performed independently by 2 authors (S.A.K. and A.A.). Prior to final inclusion, full texts of eligible articles were further screened and assessed according to methodological criteria as mentioned above.

## Results

**Fig. 2:**
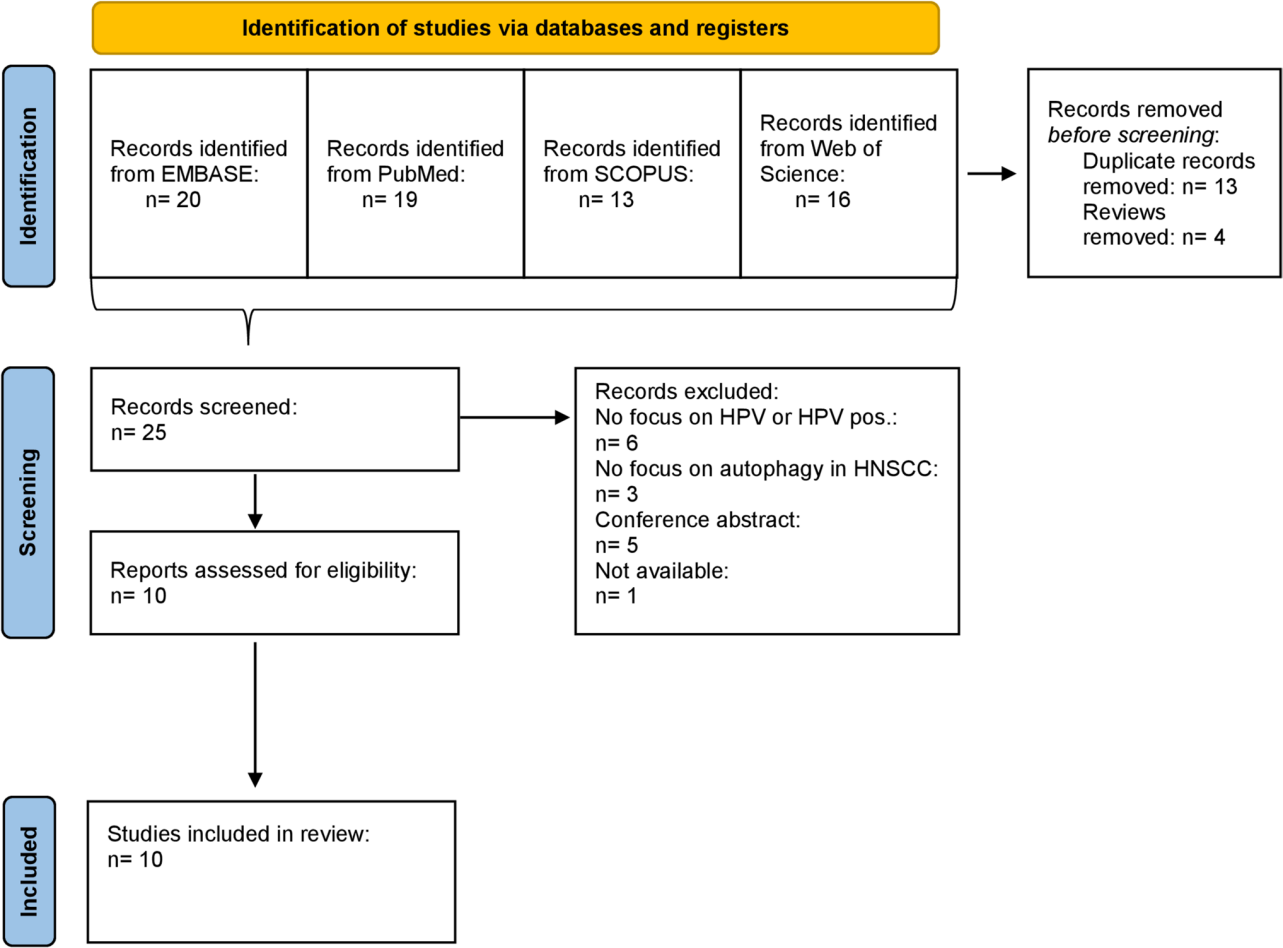
Flow diagram of the Preferred Reporting Items for Systematic Reviews and Meta-Analyses (PRISMA)

We were able to identify 68 articles after the primary screening in all databases. After eliminating duplicates and reviews, the titles, and abstracts of 25 suitable articles, published in a year range from August 2017 to February 2023, were screened. Of these, 10 full-text articles were further assessed for eligibility. 9 articles were excluded during this step due to lack of focus on HPV and/or autophagy in HNSCC. Also, 5 conference abstracts were identified and excluded as well as one paper which was not available. Ultimately, ten articles that met the inclusion criteria were included in this review (Fig. 2).

The methodology and results of each of the included studies are summarized in Table 1. Thematically, the included original articles investigated the relationship between autophagy, HPV, and HNSCC in three different settings: bioinformatic analyses, in vitro and in vivo molecular biological studies, and analyses of formalin-fixed paraffin embedded HNSCC patient samples (insert Table 1 here).

**Table 1:** Summary of the included studies, their methodologies, and results

Authors	Date	Reference#	Title	Doi	Type of article	journal	Database	Cell line	Animal	Patients		Aim	Autophagy monitoring	Results		Comments
Yang et al^a^	2020	52	Prognostic Correlation of an Autophagy-Related Gene Signature in Patients with Head and Neck Squamous Cell Carcinoma	10.1155/2020/7397132	original study	Computational and Mathematical Methods in Medicine	TCGA-HNSSC (n=495)	–	–	–		Discover correlations among 232 autophagy-associated genes from the Human Autophagy Database (ref 51) with clinicopathological features of HNSCC patients, comparative analysis between HPV+ and HPV-	–	EGFR and ITGA3 were downregulated while the protective factor CDKN2A was upregulated in HPV-positive HNSCC patients, autthors did not observe a significant correlation between HPV and prognosis of HNSCC patients		
Fang et al^a^	2021	53	Identification and validation of autophagy-related prognostic signature for head and neck squamous cell carcinoma	10.1016/j.tranon.2021.101094.	original study	Translational Oncology	TCGA-HNSSC (n= 487 for OS data; n=470 for DSS data)	–	–	–		Discover correlations among 233 autophagy-associated genes from the Human Autophagy Database (ref 51) with OS and DSS data of 487 and 470 HNSCC patients, respectively; comparative analysis between HPV+ and HPV-	–	HPV+ tumors were associated with the lower risk autophagy-associated gene risk score. Patients in low-risk score group showed higher immunescore and distinct immune cell infiltration than high-risk score group. Further analysis revealed that the CNAs of autophagy-associated gene signature affected the abundance of tumor-infiltrating immune cells		Kürten et al (ref. 86) report similar infiltration of immune cells in HPV+ HNSCC through single-RNA sequencing
Guo et al^a^	2021	54	Identification of Three Autophagy-Related Long Non-Coding RNAs as a Novel Head and Neck Squamous Cell Carcinoma Prognostic Signature	10.3389/fonc.2020.603864.	original study	Frontiers in Oncology	TCGA-HNSSC (n= not specified)	-	-	-		Discover associations between autophagy-associated lncRNAs (as defined from correlation of lncRNA expression with 232 autophagy-associated genes from the Human Autophagy Database (ref 51)) and HNSCC	-	overexpression of AL121899.1 was associated with worse prognosis, whereas overexpression of TTTY15, and MIF-AS1 correlated with better prognosis; within the HPV+ cohort, TTTY15 was overexpressed; ATG12 + BECN1 were overregulated in HPV+; MAP1LC3B downregulated in HPVpos. ATG12, BECN1 and MAP1LC3B were associated with the 3-lncRNA signature		
Thomas et al.^b^	2017	55	HPV/E7 induces chemotherapy-mediated tumor suppression by ceramide-dependent mitophagy	10.15252/emmm.201607088	original study	EMBO Molecular Medicine	–	UM-SCC-47, UM-SCC-22A, UM-SCC-1A, UPI-SCC-90	SCID mice; CerS1top/top mice	–		Investigate the role and mechanisms by which HPV signaling enhances the response of HNSCC versus cervical cancer cells to chemotherapy-mediated cellular stress by ceramide-dependent lethal mitophagy	LC3B flux assay (but without lysosomal inhibitor) + shRNA knockdown of ATG5+LC3B, TEM	HPV16 E7 activates E2F5, CDDP induces DRP1 activation, DRP1 and E2F5 make a complex and translocate to the mitochondria, enhancing ceramide-dependent mitochondrial fission. LC3B interacted with ceramide in the outer layer of the mitochondria and promoted lethal mitophagy		
Luo et al^b^	2020	57	HPV16 drives cancer immune escape via NLRX1-mediated degradation of STING	10.1172/JCI129497.	original study	Journal of Clinical Investigation	TCGA-HNSSC (n= 520)	PCI-13 and SCC90, UMSCC47 and UMSCC49, 93VU147T, FaDu, MOC2-E6/E7	Eight-week-old C57BL/6 (strain 000664, JAX), Ifnar1–/– (strain 32045-JAX), and Rag1–/– (strain 002216, JAX) mice	TMA cohort 297 HNSCC patients from the University of Michigan Head and Neck Cancer Specialized Program of Research Excellence		Elucidate the relationship of STING/IFN-I signatures and tumor-infiltrating lymphocyte (TIL) subsets in clinical HNSCC specimens and reveal the mechanisms by which HPV16 evades STING-induced IFN-I activation.	LC3B flux assay, pEGFP-LC3B laser-confocal microscopy	HPV16 E7 promotes autophagy-dependent degradation of STING, NLRX1 inhibits STING/IFN-I signaling in HPV16+ HNSCC cells; LCB-II levels were inversely correlated with STING levels; NLRX1 promoted autophagy-mediated inhibition of STING/IFN-I signaling in HPV16+ HNSCC cells. These findings were validated in an HPV16 E6/E7–expressing HNSCC C57BL/6 mouse model, MOC2-E6/E7. shNLRX1 MOC2-E6/E7 showed enhanced STING signaling.		
Antonioli et al^b^	2020	61	HPV sensitizes OPSCC cells to cisplatin-induced apoptosis by inhibiting autophagy through E7-mediated degradation of AMBRA1	10.1080/15548627.2020.1847444	original study	Autophagy	–	**HPV+**: SCC90, SCC154; **HPV-**: SCC89, SCC25; 293T cell line	–	TMA of 170 HPV+ OPSCC cases (UK COHORT; UK HPV prevalence study); TMA of 40 HPV+ OPSCC cases		Define the role of AMBRA1 in OPSCC, how HPV influences its expression and function and to explore the potential for autophagy modulation as a therapeutic strategy for OPSCC.	LC3B flux assay, LC3B-LAMP1 colocalization IF analysis	Baseline and starvation-induced autophagy flux was significantly lower in HPV16+ HNSCC cell lines compared to HPV- cells; HPV16 E7-mediated AMBRA1 degradation was reverted by calpain inhibitor ALLN		
Lee et al^b^	2021	62	p62/SQSTM1-induced caspase-8 aggresomes are essential for ionizing radiation-mediated apoptosis	10.1038/s41419-021-04301-7.	original study	Nature Cell Death and Disease	–	**HPV+:** SCC90, SCC154; **HPV-**: SCC19, WSU-HN-12 , WSU-HN-12 p62 knockdown	–	–		Elucidate the role of p62-mediated apoptosis in HNSCC	LC3B flux assay before and after radiotherapy of HNSCC cell lines	HPV16+ HNSCC cells failed to induce autophagic LC3B flux upon radiation treatments. p62 expression did not differ between HPV16+ or HPV- HNSCC cell lines. Targeting the ZZ-domain of p62 with a small molecule ligand "YOK1104" enhanced co-localization of p62 with LC3B and LAMP1 in HPV- HNSCC cells compared to HPV16+ HNSCC cells, inducing p62-dependent apoptosis via caspase-8 activation upon radiotherapy		
Cho et al^b^	2021	63	Photodynamic Therapy as a Potent Radiosensitizer in Head and Neck Squamous Cell Carcinoma	10.3390/cancers13061193.	original study	Cancers	–	**HPV+:** SCC90, SCC154;** HPV-:** SCC19, WSU-HN-12 , WSU-HN-12 p62 knockdown	–	–		Develop a mechanism-based treatment protocol for high-risk patients with HPV-negative HNSCC based on comparison of autophagic activity in HPV- and HPV+ HNSCC cells	Immunoblotting of p62 expression and LC3B flux assay before after radiotherapy (but without lysosomal inhibitor)	LC3 flux assay in fig1 is done without lysosomal protease inhibitors; no reliable autophagy monitoring making the interpretation of data regarding autophagic activity impossible.		
Shi et al^b^	2022	65	O-GlcNAcylation stabilizes the autophagy-initiating kinase ULK1 by inhibiting chaperone-mediated autophagy upon HPV infection	10.1016/j.jbc.2022.102341	original study	Journal of biological chemistry	TCGA-HNSC (n= not specified)	Human HNSCC UMSCC17B cell line with ectopic HPV16 E6, E7, E6/E7, or empty vectors expression	–	–		Describe the effects of O-GlcNAcylation in HPV+ HNSCCs.	ULK1 mass spectrometry; solely analysis on ULK1 O glcNAcylation, LC3B blotting without any flux analysis	upon HPV infection, ULK1 is hyper-O-GlcNAcylated, stabilized, and linked with autophagy elevation; ULK1 gene expression levels are elevated in HPV-positive samples, compared with HPV-negative samples; high ULK1 expression levels correlate with a better prognosis in the HPV-positive HNSCCs		
Lai et al^c^	2018	66	Differences in LC3B expression and prognostic implications in oropharyngeal and oral cavity squamous cell carcinoma patients	10.1186/s12885-018-4536-x	original study	BMC Cancer	NSW Cancer Registry	–	–	TMA of 142 cases (OPSCC=47; oral cavity=95)		Describing the difference in LC3B expression between oropharyngeal and oral cavity SCC patients through LC3B immunohistochemistry assessment and correlation with clinicopathological features of patients	LC3B immunohistochemistry	best outcomes were seen in HPV+ and low LC3B cases, and the worst in those with HPV-/high LC3B expression tumors		

^a^Rows represent bioinformatic analyses

^b^Rows represent molecular biological studies in vitro and in vivo

^c^Row represent Immunhistochemical analysis of formalin-fixed paraffin embedded HNSCC patient samples

### Bioinformatic analyses

ATG genes gained attention in the field of cancer research within the last years (Mizushima 2018). However, since numerous genes are involved directly and indirectly in the autophagic machinery, it is difficult to assign a prognostic role to a single gene (Klionsky et al. 2021a). A bioinformatic approach with comparisons between HPV^−^ and HPV^+^ subtypes and correlations with the core set of ATG genes has not been performed. However, correlations to gene signatures identified within a larger list of 232 autophagy-associated genes, which includes the ATGs (HADb; http://autophagy.lu) (Moussay et al. 2011) was performed in 3 publications.

Yang et al. (Yang et al. 2020) reported on six genes (EGFR (Epithelial growth factor receptor), HSPB8 (Heat shock protein beta 8), PRKN (Parkin), CDKN2A (cyclin-dependent kinase inhibitor 2A), FADD (Fas associated via death domain), and ITGA3 (Integrin subunit Alpha 3)) known to regulate autophagy directly or indirectly, and used these to construct a risk-signature gene cluster. This enabled the authors to stratify the patient cohort into a low-risk and high-risk group independently of clinicopathological characteristics (sex, age, clinical stage, histological grade, anatomic subdivision, alcohol history, smoking status, HPV status, and mutational status of the samples). Comparative analysis of the HPV subtypes, revealed higher expression levels of EGFR and ITGA3 in HPV^−^ HNSCC, while higher CDKN2A expression was shown in HPV^+^ HNSCC (Yang et al. 2020). Notably, a small number of HPV^+^ cases was included in this study, and it is unclear what these differences in expression levels would mean in relation to autophagy status in HPV^−^ versus HPV^+^ subtypes.

Fang et al. (2021) proposed a 14-gene autophagy-associated risk signature for the prediction of overall survival (OS) and a 12-gene risk signature for the prognosis of disease-specific survival (DSS) in HNSCC patients. They reported that the OS-related autophagy-associated gene signature revealed a high-risk score in the HPV^−^ cohort, whereas the risk score in the HPV^+^ cohort was significantly lower (Fang et al. 2021).

Lastly, Guo et al. (2020) included long non-coding RNA (lncRNA) in the construction of a prognostic risk-signature profile. After multivariate Cox regression analysis, three autophagy-associated lncRNAs were significantly associated with survival outcome: AL121899.1, TTTY15 (Testis-Specific Transcript, Y-Linked 15), and MIF-AS1 (MIF Antisense RNA 1)overexpression of AL121899.1 was associated with worse prognosis, whereas overexpression of TTTY15, and MIF-AS1 correlated with better prognosis. TTTY15 was overexpressed in the HPV^+^ HNSCC cohort. ATG12, BECN1 and MAP1LC3B (Microtubule-associated proteins 1A/1B light chain 3 B) were reported to be associated with the three autophagy-related lncRNA through analysis in Tumor Immune Estimation Resource (TIMER), Oncomine and Human Protein Atlas Database. ATG12 and BECN1 were upregulated, whilst MAP1LC3 was downregulated in HPV^+^ HNSCC tumors (Guo et al. 2020).

### Molecular biological studies in vitro and in vivo

Thomas et al. were the first to investigate the mechanisms involved in increased cell death in HPV^+^ HNSCC cells in response to chemotherapy and proposed a link to autophagy (Thomas et al. 2017). Specifically, the HPV16 E7 protein was reported enhance HNSCC cell death by selectively targeting RB (Retinoblastoma protein) and induce ceramide-dependent lethal mitophagy (Thomas et al. 2017). In a previous study of the same group, ceramide-dependent lethal mitophagy was initially suggested as a novel form of cell death in HPV^−^ HNSCC, and HPV16 E7 was identified as an activator of E2F5 (E2F Transcription Factor 5), which acts as a scaffold protein for DRP1 (Dynamin-related protein 1), an upstream inducer of ceramide-dependent lethal mitophagy (Sentelle et al. 2012). Upon DRP1 activation by Cisplatin (CDDP), the DRP1-E2F5 complex translocated to the mitochondria and enhanced ceramide-dependent mitochondrial fission (Thomas et al. 2017). This mitochondrial fission mediated an interaction between ceramide on the outer layer of the mitochondria and MAP1LC3B in the autophagosomal membrane and promoted lethal mitophagy (Thomas et al. 2017; Sentelle et al. 2012).

Another interesting interplay between autophagy and HPV16 in HNSCC was reported in a study by Luo et al. (Luo et al. 2020). It was already described that HPV18 E7 binds and inhibits STING (Stimulator of interferon genes) in HEK 293 and HeLa cells directly (Lau et al. 2015). However, it was unclear how HPV16 evades STING-induced IFN-I (Interferon-1) signaling, since HPV16 E7 might have different functions due to low degree of homology to HPV18 E7. Besides revealing a correlation between STING signaling and enhanced CD4^+^/CD8^+^ T cell infiltration, this group also noted that transfection of three HNSCC cell lines (93VU147T, UMSCC47, and FaDu) with an HPV16 E7 expression plasmid inhibited STING-induced transcription of IFN-I target genes. Interestingly, co-immunoprecipitation assays did not show any direct HPV16 E7-STING association. However, LCB-II levels were inversely correlated with STING levels in HPV16 E7-transfected HNSCC cell lines, indicating that HPV16 E7 may promote autophagy-dependent degradation of STING. A direct interaction between HPV16 E7 and NLRX1 (NOD-like receptor X1) was further investigated, since NLRX1 had been reported to potentiate autophagosome formation (Lei et al. 2012, 2016). NLRX1 promoted autophagy-mediated inhibition of STING/IFN-I signaling in HPV16^+^ HNSCC cells. These findings were validated in an HPV16 E6/E7–expressing HNSCC C57BL/6 mouse model, MOC2-E6/E7. shNLRX1 MOC2-E6/E7 showed enhanced STING signaling, leading to a decrease in cell proliferation and tumor volume, and an increase in inflammatory infiltrates within the tumor.

Another link between the HPV16 E7 protein and autophagy in HNSCC was described by Antonioli et al. (2021). Baseline and starvation-induced autophagy flux was significantly lower in HPV16^+^ HNSCC cell lines compared to HPV^−^ cells, as assessed by LC3B western blotting and LC3-LAMP1 co-localization analyses in cells that had been treated with Bafilomycin A1. It had previously been shown that HPV16 E7 mediates RB degradation in a Calpain-dependent manner (calcium-activated cysteine proteases) (Antonioli et al. 2021). Interestingly, the authors found that HPV16 E7 also mediates the degradation of AMBRA1 (Autophagy and Beclin 1 Regulator 1)—a positive regulator of autophagy—in a calpain-dependent manner, as the HPV16 E7-mediated AMBRA1 degradation could be reverted by the addition of the calpain inhibitor ALLN.

Lee et al. (2021) and Cho et al. (2021) were investigating the interplay between p62/SQSTM1 (Sequestosome 1) and HPV16 in HNSCC. Based on their experiments, Lee et al. concluded that upon radiation treatments HPV16^+^ HNSCC cells fail to induce autophagic LC3 flux, as assessed by LC3B western blotting in cells that had been treated with Bafilomycin A1, and undergo apoptotic cell death (Lee et al. 2021). Immunoblotting analyses showed no obvious difference in the expression level of p62 between HPV^−^ and HPV16^+^ cell lines. Targeting the ZZ-domain of p62 by small molecule ligand, YOK1104 (Cha-Molstad et al. 2017), induced the self-polymerization of p62 and enhanced co-localization of p62 with LC3B and LAMP1 in HPV^−^ HNSCC cells compared to HPV16^+^ HNSCC cells. Moreover, YOK1104 was able to induce p62-dependent apoptosis upon radiotherapy via caspase-8 activation (Lee et al. 2021). Cho et al. showed a similar radiosensitization upon benzoporphyrin derivative and photodynamic therapy in HPV^−^ HNSCC cells. Their study claims to show impaired autophagic activity in HPV16^+^ HNSCC cells, however, no reliable autophagy monitoring method was used to come to such conclusion. LC3B immunoblots are shown in the publication, but only before and after the application of radiotherapy. The inclusion of a lysosomal protease inhibitor is essential for the interpretation of the LC3 flux, but was not included in their experiments (Cho et al. 2021).

The most recent study regarding the interplay between HPV16 and autophagy in HNSCC, reported an increase of *O*-linked *β*-*N*-acetylglucosamine (*O*-GlcNAc) at Ser409 in ULK1 upon infection with HPV16 E6/E7-expressing lentiviral constructs (Shi et al. 2022). The Ser409 O-GlcNAc modification stabilized ULK1 protein levels. HPV16 E6/E7 expression also increased the levels of LC3-II, but it is unclear whether this was related to the increase observed in ULK1 protein levels, and unclear whether HPV16 E6/E7 expression altered autophagy activity. TCGA data analysis revealed that ULK1 mRNA is overexpressed in HPV^+^ HNSCC patient samples (which predominantly are HPV16^+^) and associated with improved overall survival.

### Formalin-fixed paraffin embedded HNSCC patient samples

In contrast to the aforementioned bioinformatics studies analyzing ATG and other autophagy-associated genes in TCGA-HNSCC data sets, only one study exclusively investigated the prognostic relevance of the expression of an autophagy-associated gene (LC3B) in HPV^+^ HNSCC in an in-house HNSCC patient cohort (Lai et al. 2018). Whereas high LC3B expression was significantly associated with poor survival and described to be an independent prognostic marker for OPSCC, this was not observed for oral SCC (although also for oral SCC, disease-free survival remained statistically significant after univariate analysis (HR = 2.36, 95% CI 1.19–4.67, *p* = 0.014), and Kaplan–Meier survival analysis showed that high LC3B expression correlated with poor overall and disease-free survival (*p* = 0.046 and 0.011, respectively). Additionally, the authors highlighted that the best outcomes were seen in HPV^+^ and low LC3B cases, and the worst in those with HPV^−^/high LC3B expression tumors. Among HPV + patients, there was a tendency of better overall survival in the low LC3B group versus the high LC3B group. It should be noted that the patient groups in these comparisons were relatively small (*n* = 8–18).

## Discussion

The role of macroautophagy in tumorigenesis and tumor progression remains incompletely understood, in part due to the challenge of accurately analyzing this dynamic process in cells and tissue. Thus, finding new ways of assessing autophagic activity and further understanding of the autophagy machinery are of outmost interest. Although autophagy has been suggested to play an important role in the development and progression of HNSCC, its role in the subtype of HPV^+^ HNSCC tumors has received less attention. Therefore, the aim of the current study was to summarize all recent evidence on the association of autophagy and HPV-infection in HNSCC.

Generally, HPV^+^ HNSCC cases are linked to an improved prognosis, mostly due to better response to radiotherapy. This fact initiated a discussion of treatment de-escalation for this patient cohort (Rühle et al. 2021; Rosenberg and Vokes 2021; Golusinski et al. 2021; Petar et al. 2021). However, a relevant subgroup of these patients has a dire prognosis, with no typical risk factors. (13) Therefore, better understanding of the tumor biology of this patient group and the influence of HPV-infection on autophagy in HNSCC is of high interest as it may facilitate discovering new therapeutic targets and prognostic markers (Brkic et al. 2022). The latter may facilitate stratification of high-risk patients and timely adaption of therapy regimens, as well as enable less aggressive treatments for low-risk patients.

In the studies included and reviewed in this work, autophagy-associated genes (including ATGs) and proteins were analyzed in bioinformatic, molecular, and clinical studies. Bioinformatic analysis in cancer research offers a new perspective in the initial search for new prognostic biomarkers and facilitates the risk-stratification prior to therapy start. Based on sequencing data of samples derived from HNSCC patients and published in “The Cancer Genome Atlas” (TCGA), numerous studies have been conducted to discover new biomarkers for HNSCC (Network 2015; Leemans et al. 2018). Some of these studies (Yang et al. 2020; Fang et al. 2021; Guo et al. 2020; Feng et al. 2020; Li et al. 2020; Ren et al. 2021; Liu et al. 2021; Jiang et al. 2021a; Zhang et al. 2022) have investigated different “autophagy-related gene signatures” comprising 232 autophagy-associated genes from the Human Autophagy Database (HADb; http://autophagy.lu) (Moussay et al. 2011), wherein three studies included comparison of HPV^+^ and HPV^−^ HNSCC (Yang et al. 2020; Fang et al. 2021; Guo et al. 2020). It should be noted that the majority of the genes within the prognostic gene signatures of each of those studies are not ATG genes, and that the 232 HADb gene list has not been updated since 2011. Additionally, the number of genes in the respective prognostic models varies greatly (2–15 genes). Interestingly, however, some common denominators can be found across several of these studies, suggesting that for instance LAMP1, GABARAPL2, and NKX2-3 expression levels may be of particularly important prognostic value in HNSCC [5475–7880]. Nonetheless, findings from bioinformatical studies investigating a potential association between clinicopathological features and so-called “autophagy-related gene signatures” have to be taken with caution. The bioinformatic studies included in this review only report gene signatures comprising genes that are either loosely connected to autophagy and/or have various functions in regulating autophagy. Furthermore, no biological validations have been considered in these studies to demonstrate biological significance. Therefore, it remains highly speculative whether the ability of the generated signatures to make clinical predictions is indeed associated with autophagy. However, similar studies are emerging rapidly, and with the development of new gene lists that increasingly integrate more updated and comprehensive knowledge, including on selective pathways (e.g., mitophagy) and, importantly, with initiatives to generate sub-lists of genes whose expression levels experimentally correlate with autophagy activity (Bordi et al. 2021) future bioinformatics analyses of patient tumor expression and clinical data will likely provide important new information on the role of autophagy in HPV^+^ and HPV^−^ HNSCC.

With regard to the increased impact of cancer immunotherapy, molecular biological investigations focusing on autophagy in relation to tumor immunology of HNSCC and the differences between HPV^+^ tumors and their negative counterpart are of particular interest.

The role of autophagy in tumor immunity is complex and incompletely understood. On one hand, autophagy can promote antigen presentation as well as differentiation, maturation and survival of immune cells in the tumor microenvironment (Zhong et al. 2016; Münz 2021; Jiang et al. 2021b). On the other hand, autophagy may alter and promote resistance of tumor cells to activated effector immune cells (Luo et al. 2020; Noman et al. 2011; Baginska et al. 2013; Messai et al. 2015). Fang et al. reported a significant association of low-risk autophagy gene signature score with high immune cell infiltration (Fang et al. 2021). At the same time, comparison between tumors with different HPV status revealed that HPV-infection is also associated with a low-risk autophagy gene signature score. This finding suggests that HPV^+^ HNSCC has a higher immune cell infiltration, but the authors did not discuss the potential immunological influence of HPV-infection in HNSCC (Fang et al. 2021). Interestingly, a higher number of tumor-infiltrating leukocytes (TILs) in HPV^+^ HNSCC tumors was reported through single-cell RNA sequencing of HNSCC patient samples (Kürten et al. 2021). Furthermore, Guo et al. described overexpression of ATG12 and BECN1 and downregulation of MAP1LC3B in HPV^+^ tumors (51). Together, these studies suggest a putative alteration in autophagy status in HPV^+^ tumors, linked with enhanced immune cell infiltration. However, interpretation of these data is limited both by the above-mentioned remarks on autophagy signature scores, and that analyses of archived tissue represent only a snapshot of the autophagic pathway. Abundance of ATG12 and BECN1 and low MAP1LC3 expression might suggest a deregulation in autophagy activity, but since there is currently not enough correlative data that solidly connect expression levels of ATGs with autophagic activity, this remains speculative. Additionally, thus far, immunohistochemical staining of ATG proteins generally lack thorough validation of antibody specificity and sensitivity (Humbert et al. 2020). Due to this current unreliability in detection of autophagic flux in paraffin-embedded tissue, the translation to clinical work-up is still missing.

Since the mechanisms of autophagy, its role in cancer and its deregulation in disease are still far from fully understood, *in* vitro and in vivo studies are essential to understand the relevance of ATG genes and respective proteins prior to translating these findings for clinical relevancy. Studies analyzing deregulation of protein function due to overexpression or knockdown/knockout of ATG genes help to elucidate the interplay between autophagy, oncoproteins, and tumor-suppressor proteins. However, correct execution and interpretation of autophagy-monitoring assays are fundamental to draw solid conclusions (Klionsky et al. 2021b). It should be noted that none of the studies included in this review have used functional, cargo-based autophagy methods, and this limits their interpretations. However, LC3 flux measurements and analysis of LC3-LAMP1 co-localization suggest that HPV16 E6/E7 proteins induce an impairment in autophagic flux (Luo et al. 2020; Antonioli et al. 2021). One mechanistic explanation to this impairment is illustrated by the finding that the HPV16 E7 protein induced the degradation of the key autophagy protein AMBRA1 via direct interaction in HNSCC cells (Antonioli et al. 2021). On the other hand, HPV16 E6/E7 proteins can induce Ser409 O-GlcNAc modification, which enhances the stability of the key autophagy-initiating protein ULK1 in HNSCC cells (51). Moreover, HPV16 E7 was reported to induce lethal mitophagy (41) as well as autophagy-dependent degradation of STING in HNSCC cells (43). It is a conceivable possibility that HPV16 infection limits some types of autophagy (as reflected by decreased LC3 flux), whilst increasing certain selective types of autophagy (e.g., of mitochondria and STING). More detailed studies that employ additional autophagy assays, including cargo-based functional assays for bulk and selective autophagy—combined with specific interference with the autophagy pathway in several different ways [see Klionsky et al. Guidelines for the use and interpretation of assays for monitoring autophagy (4th edition) (Klionsky et al. 2021b)] are required to gain more insight into how HPV16 infection affects autophagy in HNSCC cells. Studies on how HPV-infection affects autophagy in non-HNSCC cells may provide hints of general implication. Interestingly, HPV16 seems to dampen autophagy via activation of the PI3K/Akt/mTOR pathway in the early stages of virus–host cell interaction, and this limits its autophagy-mediated clearance (Surviladze et al. 2013; Griffin et al. 2013; Ishii 2013). However, additional studies are needed to elucidate the long-term effects of HPV-infection on autophagy, and the mechanisms involved.

Interestingly, an impairment in the early stages of autophagy pathway might be an underlining mechanism of lower radioresistance in HPV^+^ tumors (Digomann et al. 2019; Jing et al. 2019). Autophagy impairment may also render the tumor cells less resistant to activated effector immune cells (Luo et al. 2020; Noman et al. 2011; Baginska et al. 2013; Messai et al. 2015). This might be one of the underlining reasons of generally better prognosis of HPV^+^ HNSCC. On the other hand, autophagy impairment in tumor cells may lead to decreased antigen presentation (Zhong et al. 2016; Münz 2021; Jiang et al. 2021b) and an increased degradation of STING by HPV16 could help the HNSCC cells evade the immune system (Luo et al. 2020). Moreover, a tendency of decreased overall survival was observed in HPV^+^ HNSCC patients with high LC3B expression (52). These observations suggest that alterations in autophagy may be one of the important factors for the poor survival outcome for a sub-group of HPV^+^ HNSCC patients.

An important factor in the development of solid tumors, including HNSCC, is the interaction and communication between cancer cells and other cells of the tumor microenvironment. Of note, none of the molecular biological studies included in this review included co-culture experiments, where a possible interaction between cancer cells and stromal cells could be analyzed. Host cell autophagy can support cancer cell metabolism in a non-cell autonomous manner (Sousa et al. 2016; Katheder et al. 2017). Moreover, and of emerging interest, is the phenomenon of secretory autophagy in the co-dependency between cancer and stromal cells (New et al. 2017). The effects of HPV-infection on such interactions could be investigated in vitro by experiments on primary HNSCC cancer cells, cancer-associated fibroblasts (CAFs) and peripheral blood monocytes (PBMCs) from the same patient. Additionally, proceedings in the development of 3D cell culture have shown to be useful and closer to in vivo conditions (Ravi et al. 2015). Therefore, focusing on 3D cell culture experiments, such as patient-derived organoids, should enable faster and more reliable translation to clinical research. Taken together, these remaining challenges need to be overcome prior to successful clinical translation of autophagy research to improve translational and clinical research in this promising field.

Certain limitations of our study need to be addressed. First, the review process was conducted independently (S.A.K; A.A.)—however, titles and abstracts that do not mention the keywords of our search paradigm may have been missed. Furthermore, the inclusion criteria of this systematic review included only a small number of studies, consisting of only 10. An opportunity to broaden the study spectrum could have been to consider studies about mediators of intercellular communication in the tumor microenvironment that module the autophagy or autophagic flux inhibitors. However, this would have gone beyond the scope of our primary objective, which was to provide the first comprehensive review about the interactions of HPV and autophagy in HNSCC.

**Fig. 3:**
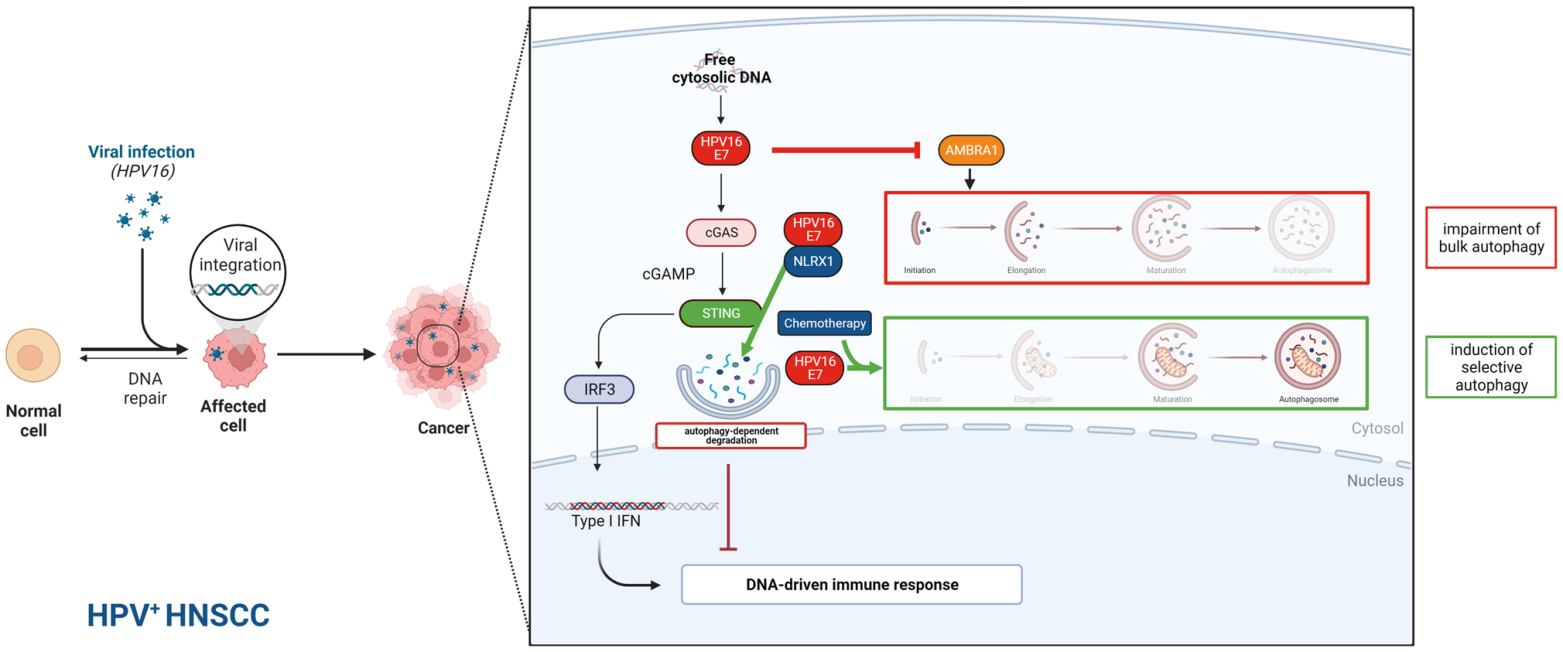
Schematic diagram of the interaction of HPV and autophagy in HNSCC. On one hand bulk autophagy may be impaired due to HPV16-E7 mediated AMBRA1 degradation. On the other hand, selective autophagy, lethal mitophagy in this case, and autophagy-dependent degradation of STING may be induced by HPV16-E7 and chemotherapy

In summary, this review highlights the significant role of HPV oncoproteins and their influence on ATGs and other autophagy-associated genes and proteins in HNSCC. Evidence suggests that bulk autophagic flux might be impaired in HPV^+^ HNSCC tumors. Moreover, the HPV16 E7 protein has been reported to induce the degradation of not only the key autophagy initiator protein AMBRA1, but also STING, which may promote tumor immune evasion despite a putative higher abundance of TILs in HPV16^+^ HNSCC. Thus, selective autophagy-dependent degradation of STING and also mitochondria may be induced by HPV16 E7 (Fig. 3). Additionally, several autophagy-associated signatures may have a prognostic significance in HPV^+^ HNSCC, although their relation to autophagy activity remains to be explored. All these findings indicate that autophagy might play a distinct role in HPV^+^ HNSCC.

## Supplementary Information

Below is the link to the electronic supplementary material.

Supplementary file1 (xlsx 13413 KB)

## Data Availability

All relevant information on data extracted of included studies are included in Table 1 within this published article. Full-text articles were downloaded from respective journals′ website and are available on request.

## Abbreviations

AJCC: American Joint Committee of Cancer
AMBRA1: Autophagy and Beclin 1 Regulator 1
ATG: Autophagy related
ATG12: Autophagy-related 12
ATG5: Autophagy-related 5
BECN1: Beclin-1
CAF: Cancer-associated fibroblast
CDDP: Cisplatin
CDKN2A: Cyclin-dependent kinase inhibitor 2A
DMBA: 7,12 Dimethylbenzanthracene
DRP1: Dynamin-related protein 1
DSS: Disease-specific survival
E2F5: E2F Transcription Factor 5
EGFR: Epithelial growth factor receptor
FADD: Fas associated via death domain
HNSCC: Head and neck squamous cell carcinoma
HPV: Human papilloma virus
HPV^−^: HPV-negative
HPV^+^: HPV-positive
HSPB8: Heat shock protein beta-8
ITGA3: Integrin Subunit Alpha 3
lncRNA: Long non-coding RNA
MAP1LC3: Microtubule-associated proteins 1A/1B light chain 3
MIF-AS1: MIF Antisense RNA 1
NLRX1: NOD-Like Receptor X1
OPSCC: Oropharyngeal squamous cell carcinoma
OS: Overall survival
p62/SQSTM1: Sequestosome-1
PRKN: Parkin
RB: Retinoblastoma-protein
STING: Stimulator of interferon genes
TCGA: The Cancer Genome Atlas
TIL: Tumor-infiltrating leukocyte
TTTY15: Testis-Specific Transcript, Y-Linked 15
ULK1: Unc51-like autophagy activating kinase 1
